# Dosing ^225^Ac-DOTATOC in patients with somatostatin-receptor-positive solid tumors: 5-year follow-up of hematological and renal toxicity

**DOI:** 10.1007/s00259-021-05474-1

**Published:** 2021-08-26

**Authors:** Clemens Kratochwil, Leonidas Apostolidis, Hendrik Rathke, Christos Apostolidis, Felix Bicu, Frank Bruchertseifer, Peter L Choyke, Uwe Haberkorn, Frederik L Giesel, Alfred Morgenstern

**Affiliations:** 1 Department of Nuclear Medicine, Heidelberg University Hospital, INF 400, 69120 Heidelberg, Germany; 2 Department of Medical Oncology, National Center for Tumor Diseases (NCT) Heidelberg, Heidelberg University Hospital, Heidelberg, Germany; 3 European Commission, Joint Research Centre (JRC), Karlsruhe, Germany; 4 Molecular Imaging Branch, National Cancer Institute, NIH, Bethesda, Maryland USA; 5 Clinical Cooperation Unit Nuclear Medicine, German Cancer Research Center (dkfz), Heidelberg, Germany; 6 Translational Lung Research Center Heidelberg (TLRC), German Center for Lung Research (DZL), Heidelberg, Germany; 7 Department of Nuclear Medicine, University Hospital Düsseldorf, Düsseldorf, Germany

**Keywords:** Targeted α therapy, TAT, Ac-225, PRRT, Neuroendocrine tumor

## Abstract

**Purpose:**

The aim of this retrospective analysis is to estimate the most appropriate single cycle and cumulative doses of ^225^Ac-DOTATOC in patients treated for somatostatin-receptor-expressing cancers.

**Methods:**

^225^Ac-DOTATOC was administered to thirty-nine patients with various somatostatin-receptor-positive tumors. Baseline and follow-up ^68^Ga-DOTATOC PET/CT, lab tests, and renal scintigraphy were obtained. Patients received long-term follow-up either at the local cancer center or in close collaboration with external oncologists. Acute and chronic hematological toxicity was evaluated quantitatively over time. Long-term follow-up of creatinine was used to approximate the annual loss of estimated GFR (eGFR).

**Results:**

Dose-dependent acute hematological toxicity was seen at single doses above 40 MBq or repeated doses greater than approximately 20 MBq ^225^Ac-DOTATOC at 4 month intervals. Treatment-related kidney failure occurred in 2 patients after a delay of >4 years but was independent of administered radioactivity, and other clinical risk factors were important contributors to renal decline. In general, the annual decline of eGFR among patients did not follow a clear dose-effect relationship even in patients with previous β-therapy. An average eGFR-loss of 8.4ml/min (9.9%) per year was observed which is similar to the experience with β-therapy studies.

**Conclusion:**

Treatment activities of approx. 20 MBq per cycle (4 monthly repetition) and cumulative doses up to 60–80 MBq generally avoided both acute and chronic grade 3/4 hematotoxicity in patients with advanced stage malignancies. Chronic renal toxicity was observed at these doses, but pre-existing renal risk factors were important co-factors. These data represent a starting point for additional research to more precisely define safety thresholds of ^225^Ac-DOTATOC.

**Supplementary Information:**

The online version contains supplementary material available at 10.1007/s00259-021-05474-1.

## Introduction

The treatment of metastatic neuroendocrine neoplasms over the past decade has undergone many changes. Conventional platin-/etoposide-based chemotherapies are only active in aggressive G3 but not G1/2 neuroendocrine tumors (NETs). The combination of temozolomide and capecitabine was suggested for NETs of pancreatic origin in 2011 [1]; the first prospective trial was recruited until 2016 (NCT01824875), but a full-text of this study is still not published. Initial phase-3 data evaluating sunitinib and everolimus in well-differentiated pancreatic NETs were published in 2011 [2, 3]; but the extended approval of everolimus for all grade 1/2 NETs of pancreatic, gastrointestinal, and lung origin only occurred in 2016 [4]. Somatostatin analogs were first approved for symptomatic treatment of carcinoid syndrome, but anti-proliferative efficacy for low proliferative (Ki-67 < 10%) enteropancreatic NETs was finally demonstrated in 2014 [5].

In contrast, peptide receptor radiotherapy (PRRT) with radiolabeled somatostatin analogs has a long history of treatment for neuroendocrine neoplasms. Promising initial results of ^111^In-Dota-lanreotid was initially reported in 1998 [6], results of ^90^Y-DOTATOC in 2001 [7], and ^177^Lu-Dotatate in 2003 [8]. Nevertheless, pivotal phase-3 evidence for well-differentiated mid-gut NETs and formal approval of ^177^Lu-Dotatate only occurred in 2017 [9]; a phase-3 study of ^177^Lu-DOTATOC in pancreatic G1/2-NETs is still recruiting (NCT03049189). Also other tumor-entities, such as paraganglioma, Merkel-cell carcinoma, small and large cell neuroendocrine carcinomas, and pheochromocytomas, can express sufficient amounts of somatostatin-receptors. However, these tumors have not yet been systematically evaluated for PRRT. Eventually, physicians considered the anti-tumor activity of β-labeled somatostatin-analogs insufficient for these more aggressive tumors.

Targeted α-radiation therapy (TAT) offers theoretical radiobiological advantages compared to β-radiation [10], but clinical long-term experience is still lacking [11]. In our hospital, ^225^Ac-DOTATOC therapies of somatostatin-receptor-positive tumors were conducted on an individual patient basis between July 2011 and March 2015. Pioneering experience with ^213^Bi-DOTATOC, which demonstrated anti-tumor activity in some patients not responding to β-PRRT [12], provided the rationale to consider ^225^Ac-TAT as an appropriate option to escalate systemic PRRT. According to then-available knowledge, we selected patients with well-differentiated gastro-entero-pancreatic (GEP)-NETs (most of our patients were diagnosed before the Ki-67-based WHO classification 2010 was introduced; today these cases would likely be classified as G1/G2 with Ki-67 <10%) for ^90^Y- or ^177^Lu-DOTATOC. ^225^Ac-DOTATOC was offered as an experimental therapy to patients with additional extra-hepatic involvement, histopathological or clinically more aggressive tumors, more advanced tumor stage, or after insufficient response to previous β-PRRT.

In this work, we evaluate the clinical follow-up data of these patients to retrospectively assess the maximum single cycle and cumulative treatment doses of ^225^Ac-DOTATOC.

## Methods

### Patients

We retrospectively analyzed all patients who were treated with ^225^Ac-DOTATOC in the Nuclear Medicine Department of Heidelberg University Hospital between July 2011 and March 2015. These patients were considered inappropriate or had already exhausted the approved treatments. Experimental salvage therapies were offered on an individual patient basis under the conditions of the updated declaration of Helsinki, paragraph 37 (Unproven Interventions in Clinical Practice). At this time, ^90^Y/^177^Lu-labeled somatostatin analogs were also still unapproved but were already often offered in well-differentiated GEP-NETs on a compassionate use basis. All patients demonstrated a positive (i.e., higher than liver background; Krenning score >2) ^68^Ga-DOTATOC PET/CT. Table 1 provides a brief overview of the analyzed patient cohort. In the supplement, we provide histopathological classification and previous therapies (supplement table 1), renal risk factors (supplement table 2), sum activity and fractioning of ^225^Ac-DOTATOC treatments, and the respective follow-up period (supplement table 3) for each patient. Patients were informed about the experimental nature of this therapy and gave written informed consent. This retrospective observational study was approved by the research ethics committee of the medical faculty of Heidelberg University (Permit S-152/2020).

**Table. 1:** Patient characteristics

Age, median (range)	60 (17–85)
No. of patients, n	39
Atypical carcinoid of the lung	5
Gastric neuroendocrine tumor	1
Pancreatic neuroendocrine tumor	8
Midgut neuroendocrine tumor	6
Hindgut neuroendocrine tumor	2
Neuroendocrine tumor of unknown primary	4
Neuroendocrine carcinoma	6
Medullary thyroid carcinoma	2
Atypical meningioma	1
Merkel cell carcinoma	1
Paraganglioma	1
Neuroendocrine prostate cancer	1
Renal neuroendocrine tumor	1
Site of metastasis, n	
Liver	32
Bone	30
Lymph node	15
Other	8
Previous therapies	
PRRT	32
SSA	21
Chemo	19
Everolimus	8
Sunitinib	6
Radioembolization	4
Interferon	2
Prior lines of systemic treatment, n	
0	2
1	12
2	8
3	5
4	8
5	3
>5	1

### Radiopharmaceuticals

^225^Ac-DOTATOC was produced in accordance with the German Pharmaceuticals Law, paragraph 13(2b). Synthesis of the peptide (DOTA0-Phe1-Tyr3)octreotide as well as radiochemical extraction of ^225^Ac from ^229^Th has already been described previously [12, 13]. For synthesis of ^225^Ac-DOTATOC, an aliquot of ^225^Ac stock solution was added to a microwave vial containing 0.1 M Tris buffer (pH 9) and an appropriate amount of DOTATOC stock solution. The reaction mixture was heated to 95 °C for 5 min using a microwave synthesizer (Biotage Initiator).

Quality control was performed by instant thin-layer chromatography using 0.05 M citric acid (pH 5) as the solvent. After development, the chromatography strip was stored for at least 1 h until radiochemical equilibrium was reached between ^225^Ac (half-life T_½_ = 9.9 d) and its daughter nuclide ^221^Fr (T_½_ = 4.8 min). Subsequently, radiochemical purity was determined by measuring the activity of the 218-keV γ-emission of ^221^Fr on the upper and lower parts of the strip using high-resolution γ-spectrometry (Ortec, AMETECH, Germany). After synthesis, an aliquot of ascorbic acid was added to the reaction mixture to minimize radiolytic degradation of ^225^Ac-DOTATOC together with an aliquot of diethyleneotriaminepentaacetic acid to scavenge free radiometals. The final pH of the formulation was 7.4. Sterility was ensured via sterile filtration.

### Treatment regimen

The treatment activity was determined based on consensus of the authors CK, UH, and FLG, who have extensive clinical experience with ^90^Y-DOTATOC, ^177^Lu-DOTATOC, and ^213^Bi-DOTATOC [12], considering the prognosis and progression velocity of each individual patient.

“Cold” somatostatin analogs were discontinued for at least 4 weeks prior to therapy. According to previous suggestions for β-PRRT, a 25 g lysine, 25 g arginine mixture (produced in the hospital’s pharmacy) [14], and 500 ml Gelafundin (Braun Melsungen) [15] were administered as a renal protective cocktail 30 min in advance and continuing until 4 h after the therapeutic i.v. injection. German radiation protection law mandates in-patient isolation on a radionuclide ward for 48 h; during this time, vital parameters and first blood sampling were obtained.

According to preclinical research that found improved renal clearance of unbound ^225^Ac decay products using chelator/diuretics therapy [16], DMPS (Dimaval Heyl, Berlin) 100 mg (morning/evening) and hydrochlorothiazide (HCT Hexal) 12.5 mg (morning) were prescribed for the first 5 days following therapy, and patients were encouraged to drink more than 2 l per day.

### Follow-up exams

Lab tests (blood cell count, creatinine, urea, and liver enzymes) were checked at baseline and then every 2 weeks until either death, change of therapy, or 6 months after the last ^225^Ac-DOTATOC therapy. Long-term survivors were then followed either in the Medical Oncology Department of our hospital or in collaboration with their local oncologist sending test results per fax; routinely these follow-up exams were done at 3–6-month intervals. Renal scintigraphy with ^99m^Tc-MAG3 (in 6 patients also per ^51^Cr-EDTA) was performed baseline, 6, and 12 months after the start of therapy following clinical routine protocols. Re-staging with ^68^Ga-DOTATOC-PET/CT was performed 3 months after each treatment cycle, respectively. In long-term follow-up, imaging was performed every 6 months using CT, MRT, or PET/CT according to the oncologist’s choice regarding medical appropriateness.

### Data analysis

Blood cell count was classified using Common Terminology Criteria for Adverse Events (CTCAE) criteria, and the non-standardized treatment activities were ordered in an ascending “quasi-escalation” manner to obtain a pseudo maximum tolerable dose. Dynamics of acute hematological toxicity over time was evaluated graphically.

To be comparable to previous literature regarding kidney follow-up after β-PRRT [17–19], the creatinine values were translated into estimates of the glomerular filtration rate (eGFR) using the MDRD formula [20]; then, using a linear regression fit, the clearance loss per year (absolute and in percentage of baseline) was approximated. Short reversible creatinine peaks, e.g., at urinary obstruction events, or as part of multiorgan failure in the last 2 weeks of life, were excluded from consideration. Only patients surviving at least 18 months after ^225^Ac-PRRT were considered relevant for the assessment of chronic kidney toxicity. Using eGFR long-term follow-up as a reference, the prognostic value of renal scintigraphy was evaluated. Other non-hematological, non-renal adverse events grade > = 2 were documented unsystematically, whenever they were considered probably or definitely treatment related.

## Results

Dose-dependent thrombocytopenia and leucopenia were the leading acute toxicities of ^225^Ac-DOTATOC. Chronic kidney disease (CKD) was the most relevant late effect in this cohort. Other adverse effects included grade 2 hair loss which was reported once. Non-TAT-specific adverse effects included nausea as a typical side effect of the kidney protective solution. Secondary myeloproliferative disease was not observed. The patient population was selected for aggressive histological sub-types and was heavily pretreated (supplement table-1). Known risk factors for CKD were found in 36/39 pts. (supplement table-2). Survival equals the follow-up period provided as the last column of supplement table-3; the median overall survival of the total cohort was 20 months.

### Single cycle dose and acute hematological toxicity

Acute toxicity was evaluable in 39 patients receiving at least one cycle of TAT. After the first administration, treatment activities of up to 44 MBq translated only into grade 0–2 events. Further escalation of treatment activity to > 45 MBq resulted in an increasing number of clinically relevant grade 3 toxicities. After the first grade 4 event was observed at around 60 MBq, no further attempts of dose escalation were done (Table 2). Both platelet and leucocyte nadirs were observed between 4 and 6 weeks p.i., followed by slow recovery to normal about 12 weeks after therapy.

**Table. 2:** Pseudo dose escalation—treatment activity vs. grading of acute hematological toxicity

Act.	Pat.	Thrombocytopenia				Leukopenia				Anemia			
MBq	n	G0/1	G2	G3	G4	G0/1	G2	G3	G4	G0/1	G2	G3	G4
<15	5	4	0	1	0	5	0	0	0	5	0	0	0
15–19	10	10	0	0	0	9	1	0	0	10	0	0	0
20–24	6	6	0	0	0	6	0	0	0	5	1	0	0
25–29	6	6	0	0	0	6	0	0	0	6	0	0	0
30–34	2	1	1	0	0	2	0	0	0	2	0	0	0
35–44	4	3	1	0	0	1	3	0	0	4	0	0	0
45–54	4	2	1	**1**	0	1	1	**2**	0	4	0	0	0
55–64	2	0	1	0	**1**	0	0	1	**1**	0	1	1	0

The events highlighted in bold definded the end-of-escalation. Thus, they are no typo but have significance as eye-catchers

### Effect of multiple cycles on myelotoxicity

**Fig. 1:**
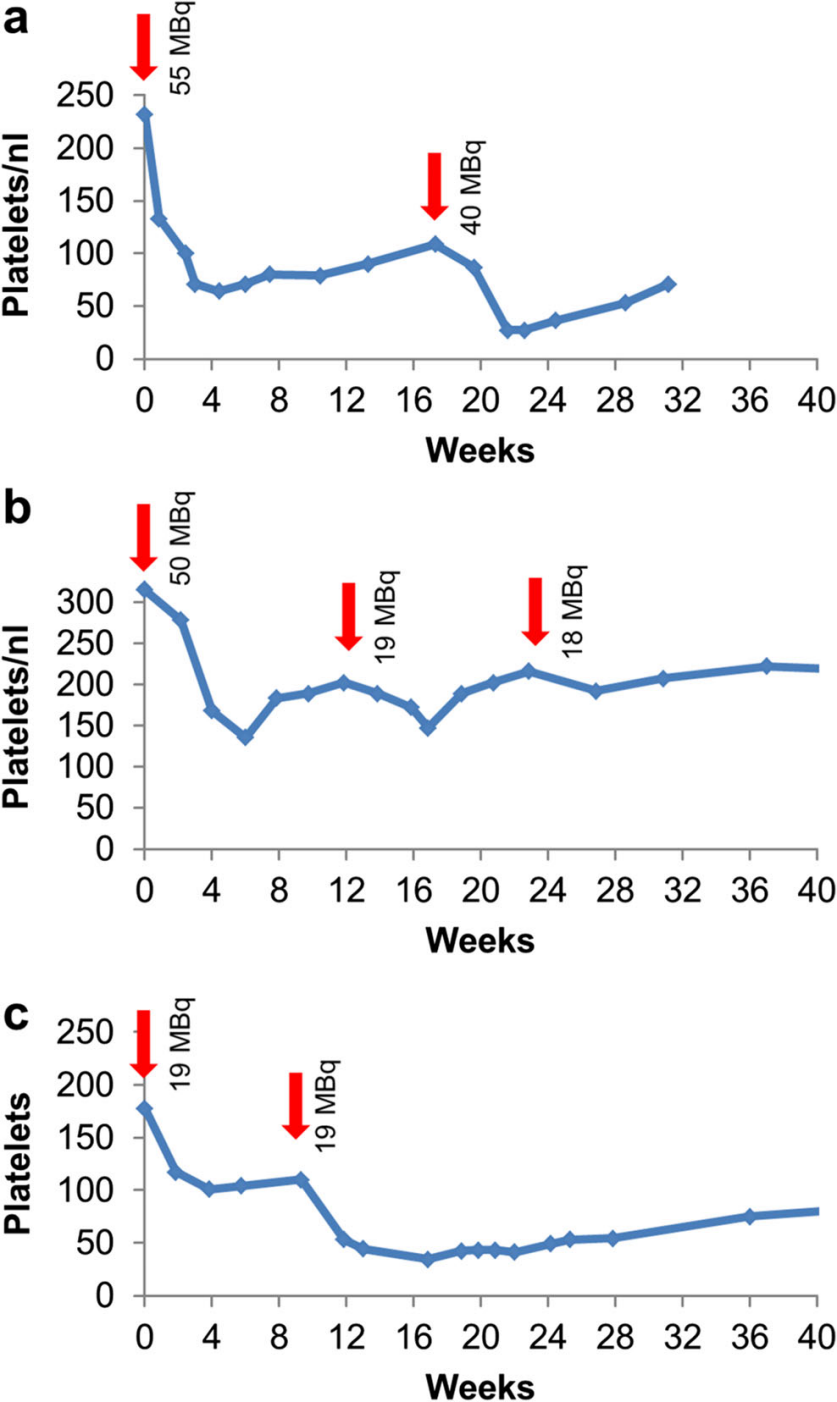
Follow-up of platelet count over time. If repeated treatments were conducted with 30–44 MBq activities, i.e., close to the maximum single dose of 45 MBq, additive toxicity occurred (**a**). In 3–4 monthly intervals, treatment activities < 25 MBq were tolerated without additive effect on thrombocytopenia (**b**). In 2 monthly interval, additive effects could even be observed for treatment activities of < 25 MBq (**c**)

Out of the 39 patients, 24 patients had a 2nd cycle, 6 patients a 3rd cycle, 2 patients a 4th cycle, and 1 patient a 5th cycle. Of the 24 patients receiving at least two therapy doses, 20 patients received their cycles in ≥ 4-month intervals; 4 patients were treated in 2–3-month intervals (Supplement Table-3). Treatment repetitions were translated into more severe acute myelosuppression when the initial treatment activity was repeated or only minimally reduced (Figure 1a). This observation indicates a need to reduce the standard treatment dose for multiple cycles below the maximum tolerated single dose. After de-escalation to treatment activities of 20–25 MBq in 4 months intervals, no additive toxicity was observed for treatment repetitions up to 5 cycles in this dosing group (Figure 1b). Among the 4 patients who were then escalated to a 2–3 monthly intervals, one patient demonstrated a clearly additive effect on platelet count (Figure 1c).

**Fig. 2:**
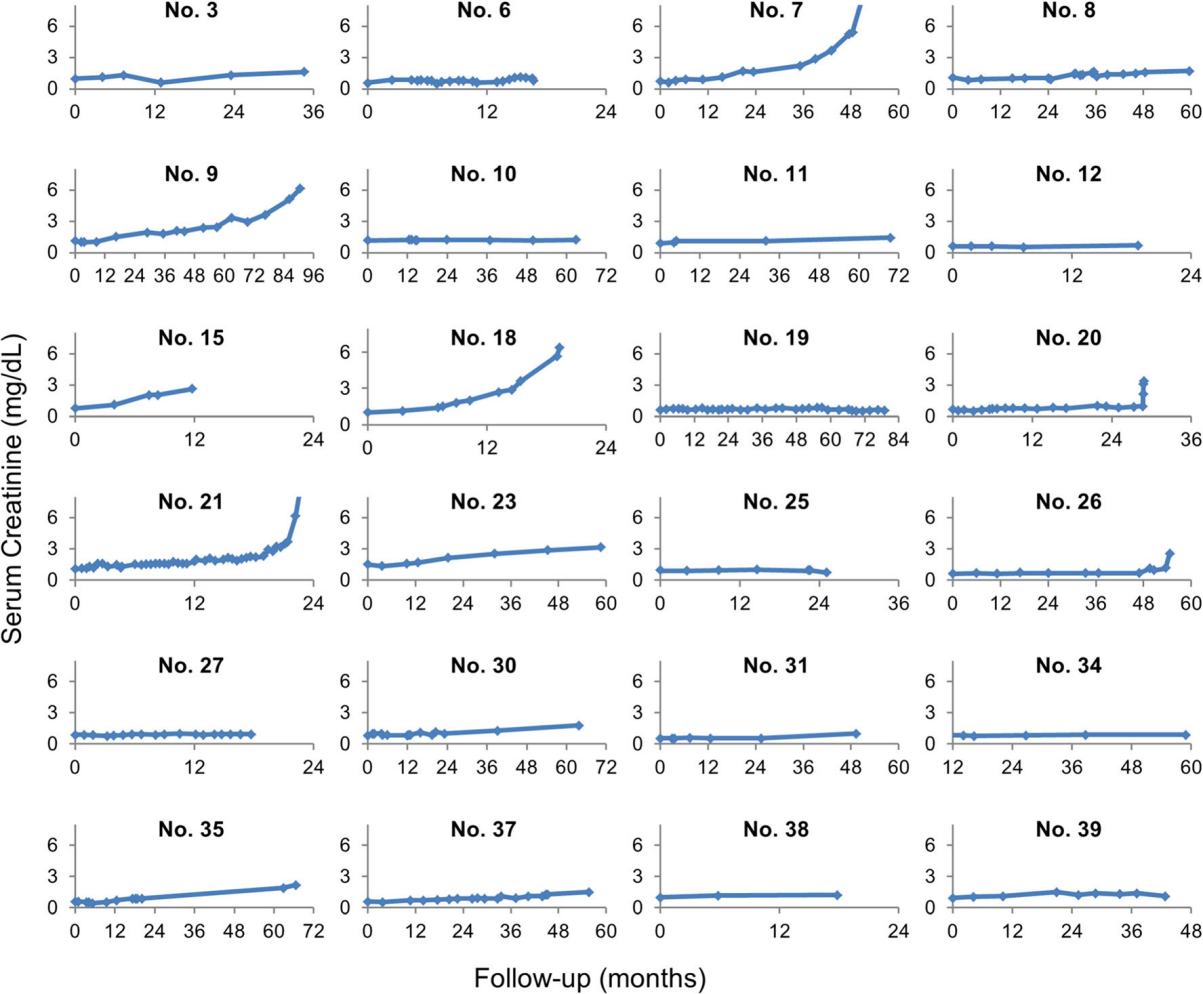
Course of serum creatinine over time of the 24 patients with the longest follow-up (patient numbers according to Supplement Table 1)

**Fig. 3:**
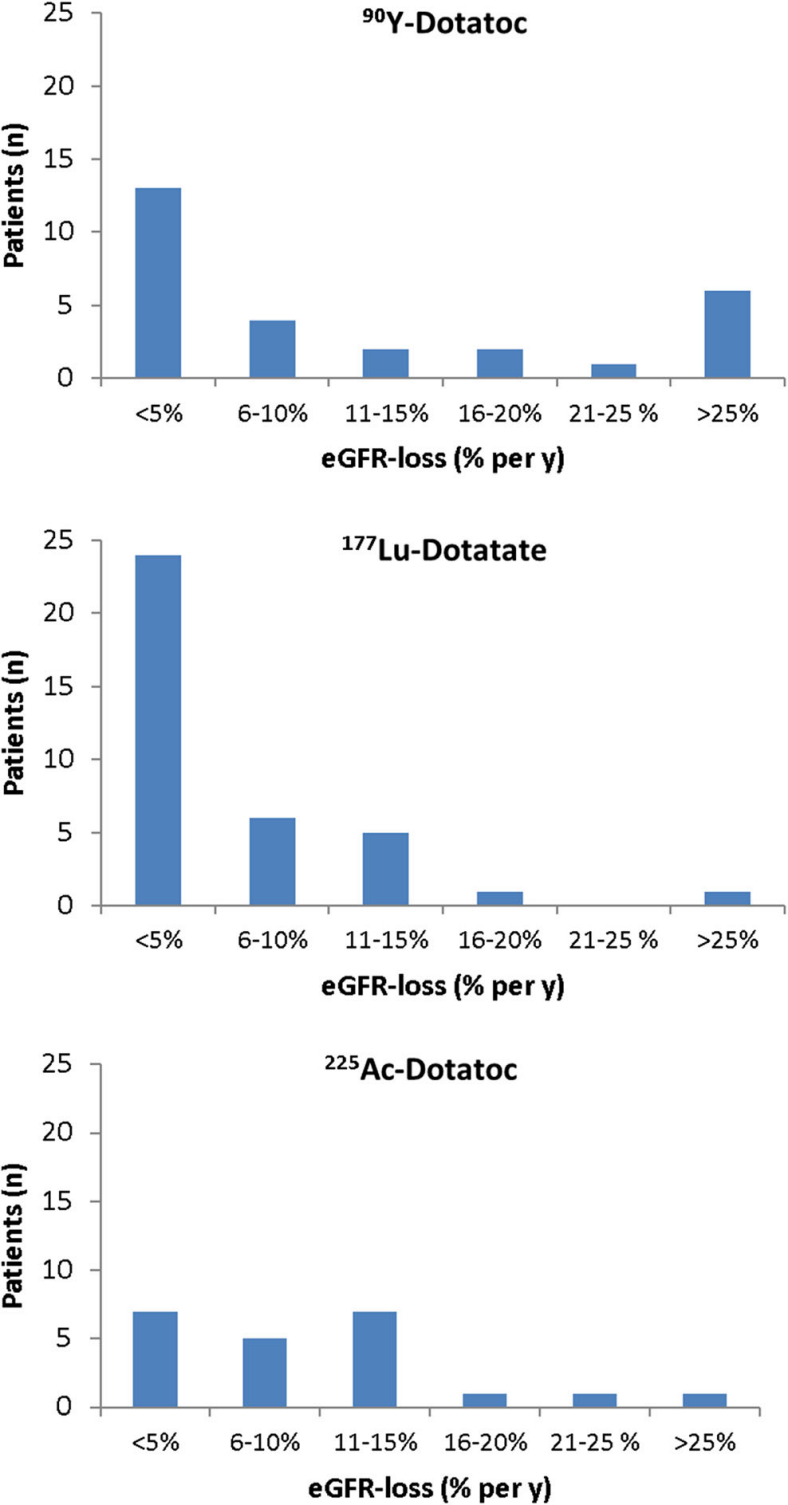
Distributions of patients to the respective extend of annual GFR-loss, following PRRT based on different radiopharmaceuticals (data for ^90^Y-DOTATOC and ^177^Lu-Dotatate are based on Ref.-17)

### Chronic renal toxicity

Of the 39 patients, 16 demonstrated tumor progression or changed therapy within < 18 month and were not suitable for evaluation of CKD. One patient was excluded due to extensive pretreatment with ^213^Bi-DOTATOC (pt. 18). For the remaining 22 patients, the median follow-up time was 57 months (range 18–90 months). The follow-up of serum creatinine (Figure 2) and the derived eGFR (Supplement Figure 1) were used to calculate an annual loss of eGFR in ml/min as a percentage of baseline. We observed an average eGFR-loss of 8.4 ml/min (9.9 % of baseline) per year. The tubular excretion rates of ^99m^Tc-MAG3 decreased by an average of 7.6% during the first 6 months and an average of 14% during the first 18 months and, thus, were well in line with the simpler serum lab tests. For not adding clinically relevant information, we also discontinued nuclear GFR measurement with ^51^Cr-EDTA (initially considered to overcome the limitations of creatinine clearance–based eGFR for values > 90 ml/min) after only 6 exams.

In addition to the mean value of renal function loss, we investigated the distribution of eGFR-losses to be comparable (Figure 3) with previous data on CKD using β-PRRT [17–19]. We observed a relatively increased fraction of 11–15% eGFR-loss per year of our TAT-treated patients although the majority of this cohort had already received β-radiation in the form of ^90^Y-DOTATOC or ^177^Lu-Dotatate.

**Fig. 4:**
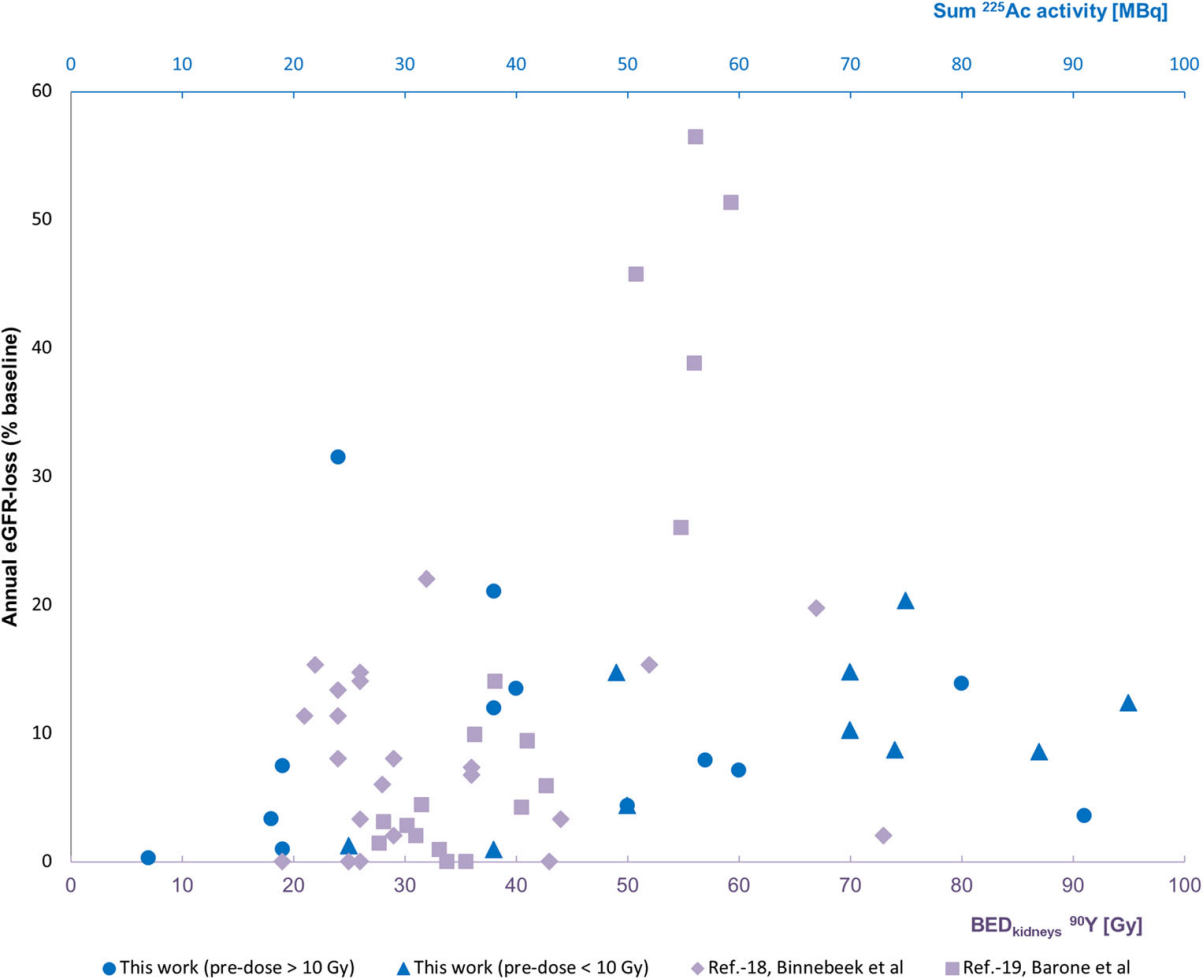
Scatter plot presenting annual GFR-loss as a function of either (lower x-axis) β-radiation absorbed dose (adopted from Ref.-18,19), overlaid with a scatter plot presenting annual GFR-loss as a function of (upper x-axis) administered activity ^225^Ac (actual data)

The scatter plot of “treatment-activity vs. annual %GFR-loss” was overlaid to literature data for “β-radiation absorbed dose vs. annual %GFR-loss” (Figure 4).

Two long-term survivors developed terminal kidney failure. One of these patients had a higher cumulative treatment activity (pt. 9, 70 MBq) and presented with 10.2% annual eGFR-loss with eGFR dropping below 10ml/min after 93 months. The second patient received a much lower cumulative treatment activity (pt. 7, 38 MBq); however, this patient had a 21.1% annual eGFR-loss, and dialysis was started after 52 months. Both patients had additional risk factors for CKD; in particular, both patients had cardiac valvular disease.

## Discussion

In this work, we elaborate on the acute hematological toxicity and chronic renal toxicity of ^225^Ac-DOTATOC therapy in patients treated between 2011 and 2015. This time frame enables consideration of 5-year follow-up in the long-term survivors of this cohort.

We found that treatment activities up to 29 MBq were hematologically tolerable (only CTCAE grade 0/1 in 26/27 patients), and severe grade 3/4 toxicities were not observed until single doses of 44 MBq were given. Repeated therapy at 4-month intervals demonstrated no additive toxicity of succeeding cycles if activities were < 25 MBq; in contrast, additive toxicity was observed in a patient treated at 2-month interval. This experience is in line with an empirical dose escalation of ^225^Ac-PSMA-617 (a PSMA-targeted agent) that was discontinued due to xerostomia, but without reaching dose-limiting hematological toxicity up to 20 MBq (200kBq/kgBW) [21]. In a recent study, ^225^Ac-DOTATATE was given in a 100kBq/kgBW dose to refractory patients or those nearing the maximum cumulative dose of ^177^Lu-DOTATATE therapy. The results were in line with our data as no grade 3/4 hematological toxicities were observed. However, the median follow-up of this study was only 8 months, and the rationale of the used dosing regimen was not fully explained [22]. The therapeutic range of a therapy is defined by the ratio of its tolerability to its anti-tumor activity. Thus, determination of a tolerable treatment without concurrent evidence for anti-tumor-activity at that dose level is inconclusive regarding the superiority of α-PRRT over β-PRRT. However, such efficacy analysis is out of the scope of this manuscript, and further efficacy studies will be required.

The mechanism of renal toxicity with either α-PRRT or β-PRRT is incompletely understood and more complex than might be thought at first consideration. Following glomerular filtration, radiolabeled peptides are re-absorbed in the tubular cells. Depending on the tissue penetration range of the emitted particle, β-emitters may reach the glomeruli, which are highly radiosensitive [23]. Some authors have speculated that the intra-renal dose distribution might be responsible for an improved tolerability of ^177^Lu-DOTATOC (max 2 mm tissue range) over ^90^Y-DOTATOC (max 12 mm) [17, 24, 25]. If this assumption about tissue range and nephrotoxicity proves true, the short range α particles emissions from ^225^Ac (approx. 0.1 mm tissue range) should result in less CKD. However, in recent studies when the biological effective dose was calculated, no inherent benefit of ^177^Lu- over ^90^Y-PRRT could be demonstrated [26, 27]. In one preclinical model, glomerulopathy but no tubular degeneration was observed with β-emitting ^177^Lu-DOTA-PESIN; in contrast, tubular degeneration was the leading pathology observed for α-emitting ^213^Bi-DOTA-PESIN [28]. In another preclinical work, α-camera images revealed “hotspots” of ^211^At-PSMA only in the proximal convoluted tubules of the kidney, but this was leading to a generally reduced function of the whole nephron. [29]. Thus, the benefit of the “range effect” may be overestimated in importance when considering nephrotoxicity. Renal toxicity may be further complicated by the fate of nuclide-specific daughter isotopes that may cause additional renal damage [16]. For instance, when ^213^Bi (a daughter isotope of ^225^Ac) becomes unbound from the targeting moiety while it is still in circulation, it can accumulate in the kidneys [30]. However, after ^225^Ac-TAT internalizes within a cell, the daughter nuclides will be generated intra-cellularly. The latter mechanism is favored with ^225^Ac DOTATOC because somatostatin-receptor agonists induce receptor internalization [31] and have rapid blood clearance [32]. Nevertheless, daughter nuclide translocation remains an uncertainty factor regarding accurate dosimetry estimates for the kidney. Indeed, due to the lack of practical α-dosimetry software tools, reliable organ-specific RBE values (relative biological efficacy), and established models for micro-dosimetry and translocation effects, personalized dosimetry estimates have not been reasonable for the presented patients.

**Fig. 5:**
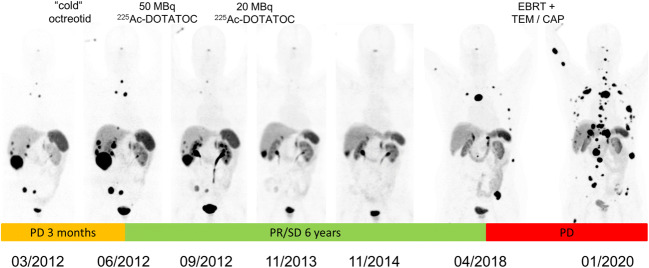
A patient who presented with progression of disease (size and novel lesions) during only 3 months of somatostatin analog therapy (orange time-frame), demonstrated enduring tumor response to ^225^Ac-DOTATOC for nearly 6 years (green). Next progression was treated with external-beam radiotherapy of painful bone lesions and temozolomide/capecitabine, however again with only short benefit to conventional therapy (red)

As demonstrated in Figure 4, we did not observe a correlation between treatment activity and nephrotoxicity, with or without prior exposure to β therapies. The relatively lower fraction of patients who developed < 5% eGFR-loss per year compared to ^177^Lu-Dotatate (Figure 3) can probably be related to the fact that the historical controls were typically radiation-naïve while the majority of our patients had already had β-PRRT and therefore pre-existing a reduced tolerance reserve. Most importantly in both subgroups, we rarely observed more than 20% eGFR-loss per year. At this rate of decline, approximately 5 years must elapse between treatment and the requirement for dialysis, assuming normal kidney function at baseline. Indeed, the kidney failure that was finally observed in two of our patients was a result of moderate but persistent annual eGFR-loss over more than 4 years. Taking into account the limited duration of response to other treatments, α-PRRT probably contributed to a remarkable prolonged survival of these patients. Elsewise they would have likely succumbed to their disease before incurring renal toxicity (Figure 5). Renal scintigraphy was not helpful in identifying such patients in advance. This agrees with observations with β-PRRT [33]. However, renal scintigraphy is still useful to rule out urinary obstruction in advance of radionuclide therapy in selected patients.

Several years ago, a small molecule targeting prostate-specific membrane antigen (PSMA) with tumor uptake and clearance kinetics similar to DOTATOC was developed and labeled with ^225^Ac. Recently, a first patient treated with that agent survived 5 years after therapy with 24 MBq ^225^Ac-PSMA-617, accompanied by a slow but chronic increase of serum creatinine [34]. Another two patients that were treated with activities of 20–24 MBq ^225^Ac-PSMA-617 developed chronic kidney disease, and biopsy revealed both glomerular and tubular pathologies in one of them [35]. All three patients had more than one additional risk factor for occurrence of CKD (age, diabetes, hypertension, etc.). However, the dose difference between the reports of renal toxicity after 24 MBq ^225^Ac-PSMA-617 and the moderate renal toxicity we observed after up to cumulative 95 MBq ^225^Ac-DOTATOC is remarkable. One explanation could be that despite comparable dose at the whole-organ level, ligand-specific micro-dosimetry might be a significant issue when α-emitters are used. In addition to its nonspecific excretory function, the kidneys also have physiological expression of somatostatin-receptors and PSMA [29, 36]. While PSMA is expressed in neoangiogenesis, renal somatostatin-receptors may play a role in a pro-inflammatory cascade since overexpression has been found in IgE-nephropathy [37]. Highly focused α-radiation to somatostatin-receptor-positive cells may induce a different renal remodeling response than targeting PSMA-positive cells.

There are various reasons for the absence of a dose response relationship for radiotoxicity of the kidney. Even in healthy individuals, there is a physiological decline of kidney function of 10ml/min per decade. In addition, risk factors for CKD are common, and the incidence of kidney failure normally affects patients who were never treated with radioactive drugs. In well-differentiated NETs, additional tumor-related mechanisms are frequent, e.g., hepatorenal syndrome caused by liver metastases, pre-renal azotemia (e.g., diarrhea as part of the carcinoid syndrome), or post-renal (e.g. right heart failure associated with carcinoid heart syndrome); in aggressive tumors, often potentially nephrotoxic chemotherapies are used.

Some centers strictly following the recommended kidney tolerance thresholds and not exceeding 4 × 7.4 GBq ^177^Lu-Dotatate reported either no grade 3 or 4 subacute nephrotoxicity in 323 patients [38] or only 1.5% grade 3 or 4 nephrotoxicity in 807 patients [39]. In line with our observations, in these studies, the kidney radiation absorbed dose did not correlate with renal function loss over time. These results suggest higher doses with better efficacy may be possible. Thus, in our center, the general concept is to administer relatively conservative doses to early stage patients and accept higher risks in later-stage or more aggressive tumors. However, if ^225^Ac-DOTATOC would be considered for first-line therapy of well-differentiated NETs with a prognosis > 5 years, a 10–15% decline of GFR per year would be considered unacceptable, and lower treatment activities should be administered. We suggest that a 4 × 10 MBq schema would be more appropriate for such patients.

This study has several inherent limitations. The study was retrospective, and treatment doses were defined on a case by case basis—explicitly considering recent lab tests and the patient’s individual prognosis, influenced by total tumor volume, site of metastases, and growth velocity. This naturally leads to selection biases. Patients who were considered to be in a more vulnerable general condition received lower treatment activities than patients who presented in better physical condition. The so-called tumor sink effect, i.e., the observation that normal organ uptake is inversely correlated to the total tumor volume [40, 41], is probably also a relevant confounder. In our cohort, the highest activities were selectively prescribed to patients with high tumor burden. A relevant fraction of the injected radiopharmaceutical was likely trapped in well perfused high-volume tumor lesions, thus accelerating blood-pool clearance and lowering red-marrow dose and the fraction of drug that has to be cleared by the kidneys. These two factors tend to overestimate the risk of lower treatment doses and the tolerability of higher treatment doses. The heterogeneous nature of the patient population affects standardized evaluation. As an example, inclusion of several different tumor entities increases the diversity of previous therapies, which each having diverging nephrotoxic potential making conclusions even more difficult. However, for ethical reasons, potentially irreversible, sometimes lethal and often late occurring side effects such as chronic kidney disease cannot systematically be studied in homogeneous cohorts of early stage patients. Thus, in our opinion, any new information that might positively contribute to patient safety in the future with regard to the design of clinical trials is worth sharing with the community.

## Conclusion

Although α-emitters offer potential advantages as radiopharmaceutical therapies and have been used in small studies, the availability of long-term toxicity data is lacking. We found treatment activities of approx. 20 MBq per cycle (4 monthly repetition) and cumulative doses up to 60–80 MBq ^225^Ac-DOTATOC reasonable for patients with advanced stage malignancies. This kind of data may be useful when planning prospective clinical trials using this agent or other related therapies based on α-emitters.

## Supplementary Information

ESM 1 (PDF 120 kb)

ESM 2 (PNG 531 kb)

High resolution image (TIF 780 kb)

## Declarations

### Clinical trial registration

We retrospectively analyzed data, which were generated in clinical practice, but not an interventional trial. No registration is needed for this kind of study.

## References

[1] Strosberg JR, Fine RL, Choi J, Nasir A, Coppola D, Chen DT, Helm J, Kvols L (2011). First-line chemotherapy with capecitabine and temozolomide in patients with metastatic pancreatic endocrine carcinomas. Cancer..

[2] Raymond E, Dahan L, Raoul JL (2011). Sunitinib malate for the treatment of pancreatic neuroendocrine tumors. N Engl J Med.

[3] Yao JC, Shah MH, Ito T (2011). Everolimus for advanced pancreatic neuroendocrine tumors. N Engl J Med.

[4] Yao JC, Fazio N, Singh S (2016). Everolimus for the treatment of advanced, non-functional neuroendocrine tumours of the lung or gastrointestinal tract (RADIANT-4): a randomised, placebo-controlled, phase 3 study. Lancet..

[5] Caplin ME, Pavel M, Ćwikła JB (2014). Lanreotide in metastatic enteropancreatic neuroendocrine tumors. N Engl J Med.

[6] Virgolini I, Szilvasi I, Kurtaran A (1998). Indium-111-DOTA-lanreotide: biodistribution, safety and radiation absorbed dose in tumor patients. J Nucl Med.

[7] Waldherr C, Pless M, Maecke HR, Haldemann A, Mueller-Brand J (2001). The clinical value of [90Y-DOTA]-D-Phe1-Tyr3-octreotide (90Y-DOTATOC) in the treatment of neuroendocrine tumours: a clinical phase II study. Ann Oncol.

[8] Kwekkeboom DJ, Bakker WH, Kam BL (2003). Treatment of patients with gastro-entero-pancreatic (GEP) tumours with the novel radiolabelled somatostatin analogue [177Lu-DOTA(0),Tyr3]octreotate. Eur J Nucl Med Mol Imaging.

[9] Strosberg J, El-Haddad G, Wolin E (2017). Phase 3 Trial of 177Lu-Dotatate for midgut neuroendocrine tumors. N Engl J Med.

[10] Sgouros G (2020). Dosimetry, Radiobiology and synthetic lethality: radiopharmaceutical therapy (RPT) with alpha-particle-emitters. Semin Nucl Med.

[11] Morgenstern A, Apostolidis C, Kratochwil C, Sathekge M, Krolicki L, Bruchertseifer F (2018). An overview of targeted alpha therapy with 225Actinium and 213Bismuth. Curr Radiopharm.

[12] Kratochwil C, Giesel FL, Bruchertseifer F (2014). ^213^Bi-DOTATOC receptor-targeted alpha-radionuclide therapy induces remission in neuroendocrine tumours refractory to beta radiation: a first-in-human experience. Eur J Nucl Med Mol Imaging.

[13] Apostolidis C, Molinet R, Rasmussen G, Morgenstern A (2005). Production of Ac-225 from Th-229 for targeted alpha therapy. Anal Chem.

[14] Rolleman EJ, Valkema R, de Jong M, Kooij PP, Krenning EP (2003). Safe and effective inhibition of renal uptake of radiolabelled octreotide by a combination of lysine and arginine. Eur J Nucl Med Mol Imaging.

[15] Vegt E, Wetzels JF, Russel FG, Masereeuw R, Boerman OC, van Eerd JE, Corstens FH, Oyen WJ (2006). Renal uptake of radiolabeled octreotide in human subjects is efficiently inhibited by succinylated gelatin. J Nucl Med.

[16] Jaggi JS, Kappel BJ, McDevitt MR, Sgouros G, Flombaum CD, Cabassa C, Scheinberg DA (2005). Efforts to control the errant products of a targeted in vivo generator. Cancer Res.

[17] Valkema R, Pauwels SA, Kvols LK (2005). Long-term follow-up of renal function after peptide receptor radiation therapy with (90)Y-DOTA(0),Tyr(3)-octreotide and (177)Lu-DOTA(0), Tyr(3)-octreotate. J Nucl Med.

[18] Van Binnebeek S, Baete K, Vanbilloen B (2014). Individualized dosimetry-based activity reduction of ^90^Y-DOTATOC prevents severe and rapid kidney function deterioration from peptide receptor radionuclide therapy. Eur J Nucl Med Mol Imaging.

[19] Barone R, Borson-Chazot F, Valkema R (2005). Patient-specific dosimetry in predicting renal toxicity with (90)Y-DOTATOC: relevance of kidney volume and dose rate in finding a dose-effect relationship. J Nucl Med.

[20] Levey AS, Coresh J, Greene T, Marsh J, Stevens LA, Kusek JW (2007). Van Lente F; Chronic Kidney disease epidemiology collaboration. Expressing the modification of diet in renal disease study equation for estimating glomerular filtration rate with standardized serum creatinine values. Clin Chem.

[21] Kratochwil C, Bruchertseifer F, Rathke H (2017). Targeted α-therapy of metastatic castration-resistant prostate cancer with ^225^Ac-PSMA-617: dosimetry estimate and empiric dose finding. J Nucl Med.

[22] Ballal S, Yadav MP, Bal C, Sahoo RK, Tripathi M (2020). Broadening horizons with ^225^Ac-DOTATATE targeted alpha therapy for gastroenteropancreatic neuroendocrine tumour patients stable or refractory to ^177^Lu-DOTATATE PRRT: first clinical experience on the efficacy and safety. Eur J Nucl Med Mol Imaging.

[23] Sharma M, McCarthy ET, Sharma R, Fish BL, Savin VJ, Cohen EP, Moulder JE (2006). Arachidonic acid metabolites mediate the radiation-induced increase in glomerular albumin permeability. Exp Biol Med (Maywood).

[24] Konijnenberg M, Melis M, Valkema R, Krenning E, de Jong M (2007). Radiation dose distribution in human kidneys by octreotides in peptide receptor radionuclide therapy. J Nucl Med.

[25] Bodei L, Cremonesi M, Ferrari M (2008). Long-term evaluation of renal toxicity after peptide receptor radionuclide therapy with 90Y-DOTATOC and 177Lu-DOTATATE: the role of associated risk factors. Eur J Nucl Med Mol Imaging.

[26] Romer A, Seiler D, Marincek N (2014). Somatostatin-based radiopeptide therapy with [177Lu-DOTA]-TOC versus [90Y-DOTA]-TOC in neuroendocrine tumours. Eur J Nucl Med Mol Imaging.

[27] Radojewski P, Dumont R, Marincek N, Brunner P, Mäcke HR, Müller-Brand J, Briel M, Walter MA (2015). Towards tailored radiopeptide therapy. Eur J Nucl Med Mol Imaging.

[28] Wild D, Frischknecht M, Zhang H (2011). Alpha- versus beta-particle radiopeptide therapy in a human prostate cancer model (213Bi-DOTA-PESIN and 213Bi-AMBA versus 177Lu-DOTA-PESIN). Cancer Res.

[29] Kiess AP, Minn I, Vaidyanathan G (2016). (2S)-2-(3-(1-Carboxy-5-(4-211At-Astatobenzamido)Pentyl)Ureido)-pentanedioic acid for psma-targeted α-particle radiopharmaceutical therapy. J Nucl Med.

[30] Schwartz J, Jaggi JS, O'Donoghue JA, Ruan S, McDevitt M, Larson SM, Scheinberg DA, Humm JL (2011). Renal uptake of bismuth-213 and its contribution to kidney radiation dose following administration of actinium-225-labeled antibody. Phys Med Biol.

[31] Cescato R, Schulz S, Waser B (2006). Internalization of sst2, sst3, and sst5 receptors: effects of somatostatin agonists and antagonists. J Nucl Med.

[32] Koukouraki S, Strauss LG, Georgoulias V, Schuhmacher J, Haberkorn U, Karkavitsas N, Dimitrakopoulou-Strauss A (2006). Evaluation of the pharmacokinetics of 68Ga-DOTATOC in patients with metastatic neuroendocrine tumours scheduled for 90Y-DOTATOC therapy. Eur J Nucl Med Mol Imaging.

[33] Werner RA, Beykan S, Higuchi T (2016). The impact of 177Lu-octreotide therapy on 99mTc-MAG3 clearance is not predictive for late nephropathy. Oncotarget.

[34] Rathke H, Bruchertseifer F, Kratochwil C, Keller H, Giesel FL, Apostolidis C, Haberkorn U, Morgenstern A (2021). First patient exceeding 5-year complete remission after ^225^Ac-PSMA-TAT. Eur J Nucl Med Mol Imaging.

[35] Pelletier K, Côté G, Fallah-Rad N, John R, Kitchlu A (2020). CKD After 225Ac-PSMA617 therapy in patients with metastatic prostate cancer. Kidney Int Rep.

[36] Boy C, Heusner TA, Poeppel TD (2011). 68Ga-DOTATOC PET/CT and somatostatin receptor (sst1-sst5) expression in normal human tissue: correlation of sst2 mRNA and SUVmax. Eur J Nucl Med Mol Imaging.

[37] Bhandari S, Watson N, Long E, Sharpe S, Zhong W, Xu SZ, Atkin SL (2008). Expression of somatostatin and somatostatin receptor subtypes 1-5 in human normal and diseased kidney. J Histochem Cytochem.

[38] Bergsma H, Konijnenberg MW, van der Zwan WA, Kam BL, Teunissen JJ, Kooij PP, Mauff KA, Krenning EP, Kwekkeboom DJ (2016). Nephrotoxicity after PRRT with (177)Lu-DOTA-octreotate. Eur J Nucl Med Mol Imaging.

[39] Bodei L, Kidd M, Paganelli G, Grana CM, Drozdov I, Cremonesi M, Lepensky C, Kwekkeboom DJ, Baum RP, Krenning EP, Modlin IM (2015). Long-term tolerability of PRRT in 807 patients with neuroendocrine tumours: the value and limitations of clinical factors. Eur J Nucl Med Mol Imaging.

[40] Filss C, Heinzel A, Miiller B, Vogg ATJ, Langen KJ, Mottaghy FM (2018). Relevant tumor sink effect in prostate cancer patients receiving 177Lu-PSMA-617 radioligand therapy. Nuklearmedizin..

[41] Beauregard JM, Hofman MS, Kong G, Hicks RJ (2012). The tumour sink effect on the biodistribution of 68Ga-DOTA-octreotate: implications for peptide receptor radionuclide therapy. Eur J Nucl Med Mol Imaging.

