# Supplementary material for: Dosing 225Ac-DOTATOC in patients with somatostatin-receptor-positive solid tumors: 5-year follow-up of hematological and renal toxicity
Source: Eur J Nucl Med Mol Imaging. 2021 Aug 26;49(1):54–63. doi: 10.1007/s00259-021-05474-1 (PMC8712294; doi:10.1007/s00259-021-05474-1)
Supplement: Supplementary file 1 — (PDF 120 kb) [file 259_2021_5474_MOESM1_ESM.pdf]

Supplement Table 1: Detailed patient characteristics

Pt.-No.	Age	Histological Diagnosis	Metastasis (UICC abbr.)	Previous Pharmacological Therapies *(italicized: after TAT, during kidney follow-up)*	Previous PRRT Y-90/ Lu-177/ Bi-213 [GBq]
1	85	Gastric-NET G2 (Ki67 10%)	hep, oss		8 / 8 / 0
2	60	Gastric-NEC G3 (Ki67 30%)	hep, oss	Carbo/Eto	6 / 12 / 0
3	62	Midgut-NET	hep, oss	SSA	0 / 0 / 0
4	73	Midgut-NET	lym, oss, hep	SSA, Eve	16 / 20 / 0
5	53	Midgut-NET	lym, per	SSA	0 / 0 / 0
6	58	Midgut-NET G2 (Ki67 15%)	lym, hep, oss, per	SSA, INF	0 / 0 / 0
7	65	Midgut-NET G2 (Ki67 15%)	lym, hep, oss	SSA, Deno	6 / 4 / 0
8	73	Midgut-NET G2 (Ki-67 6%)	lym, hep, oss	SSA, Deno	12 / 0 / 0
9	66	Hindgut-NET	hep, oss	SSA, *Tem/Cap*	0 / 0 / 0
10	52	Hindgut-NET G2 (10%)	lym, hep, oss		10 / 6 / 0
11	56	p-NET	hep, oss, lym		0 / 15 / 0
12	49	p-NET	lym, hep, oss	SSA, STZ/Doxo, Sun, Eve, Oxa/5-FU, SIRT	11 / 1 / 0
13	56	p-NET	hep, oss	SSA, Tem/Cap, Sun, Eve	0 / 44 / 0
14	55	p-NET G2 (Ki-67 10%)	oss, hep	SSA, Eve, SIRT, STZ/5-FU	12 / 20 / 0
15	61	p-NET G2 (Ki-67 10%)	hep, oss, adr	SSA	22 / 12 / 13
16	56	p-NET G2 (Ki-67 15%)	lym, oss, hep	STZ/5-FU,	6 / 24 / 0
17	64	p-NET G2 (Ki67 18%)	hep, oss, pulm	STZ/5-FU, Eve, Sun	8 / 6 / 0
18	69	p-NET G2(Ki-67 18%)	lym, hep	SSA, Sun	18 / 4 / 19
19	17	p-NET G3 (Ki-67 30%)	lym, hep, per	Tem/Thal, SSA, Sun	6 / 19 / 0
20	47	p-NET G3 (Ki-67 30%)	hep	SSA, *STZ/5-FU, Tem/Cap/Bev, Cis/Eto, Eve, FOLFIRI/Bev/Nivo*	6 / 12 / 0
21	73	p-NEC (Ki-67 25%)	hep, oss	Cis/Eto, Topo, SIRT	10 / 4 / 0
22	47	p-NEC (Ki-67 30%)	hep, oss		6 / 4 / 0
23	67	atyp lung carcinoid	hep	SSA	2 / 12 / 0
24	34	atyp lung carcinoid (Ki-67 10%)	hep, oss	SSA, Doxo/Vincri/Cyclo, Eve	14 / 16 / 15
25	65	atyp lung carcinoid (Ki-67 10%)	lym, hep, oss	Carbo/Eto, SIRT	0 / 30 / 0
26	58	atyp lung carcinoid (Ki-67 20%)	oss	SSA	4 / 0 / 0
27	72	atyp lung carcinoid (Ki-67 8%)	hep, oss		0 / 0 / 4
28	72	Lung-LCNEC (Ki-67 60%)	lym, oss, hep		14 / 6 / 0
29	51	CUP-NET G2 (Ki-67 15%)	lym, hep, oss, oth	SSA, TACE, FOLFOX, Eve, *Sun*	16 / 8 / 0
30	63	CUP-NET G1	oss, hep		0 / 0 / 0
31	58	CUP-NET G2	lym, hep, oss		0 / 18 / 0
32	71	CUP-NET G3 (Ki-67 25%)	hep, oss	SSA	6 / 14 / 0
33	61	Medullary thyroid carcinoma	oss	Van	0 / 0 / 0
34	52	Medullary thyroid carcinoma	pulm, oss		0 / 6 / 0
35	55	Meningeoma atyp. (WHO II)			0 / 0 / 0
36	38	Merkel-cell carcinoma	pulm, pleu, per	Carbo/Eto, Pac, Ave	3 / 15 / 0
37	40	Paraganglioma	oss, oth	Cis/Eto, Cyclo/Vincri/DTIC, Sun	2 / 2 / 0
38	66	Prostate-NET (Ki-67 10%)	hep, oss	SSA, Eve, Deno	6 / 12 / 4
39	42	Renal-NET G2 (Ki-67 5%)	lym, hep, oss	SSA, INF, Deno	0 / 22 / 0

SSA Somatostatin-Analogue, Eve Everolimus, Sun Sunitinib, STZ Streptozocin, 5-FU 5-Flurouracil, Doxo Doxorubicin, Tem Temozolomide, Cap Capecitabine, Thal Thalidomide, Cis Cisplatin, Carbo Carboplatin, Oxa Oxaliplatin, Eto Etoposide, Topo Topotecan, Vincri Vincristin, Cyclo Cyclophosphamide, Deno Denosumab, IFN Interferon, SIRT Selective Internal Radiotherapy, Van Vandetanib, Ave Avelumab, Pac Paclitaxel, DTIC Dacarbacin, FOLFOX Oxa+5-FU, , FOLFIRI Irinotecan+5-FU, TACE Trans-Arterial Chemo-Embolization; NET Neuroendocrine Tumor, NEC Neuroendocrine Carcinoma, CUP Carcinoma of unknown primary, p-NET pancreatic NET

Supplement Table 2: Individual risk-factors regarding Chronic Kidney Disease (CKD)

Pt.-No.	Gender	CKD risk factors	Estimated kidney pre-dose [Gy]*
1	f	age >65, diabetes, hypertension, bisphosphonates	22
2	m	CVD (septum defect), hypertension, ntCTX	21
3	m	hypertension	0
4	m	age >65, hypertension, carcinoid syndrom, targetedCTX	48
5	m	carcinoid syndrom	0
6	f	diabetes, hypertension, BMI >30, NSAID	0
7	f	age >65, CVD (tricuspidal+mitral insuff.), carcinoid syndrom, Deno	15
8	m	age >65, CVD (tricuspidal+mitral replacement), carcinoid syndrom, hypertension, diabetes, Deno	25
9	m	age >65, CVD (ICD, infarction, EF 43%), hypertension	0
10	m	hypertension	25
11	m	-	11
12	f	CVD (septum defect), ntCTX	24
13	m	ntCTX, targetedCTX	31
14	f	ntCTX (breast cancer), bisphosphonates	39
15	f	bisphosphonates	77
16	f	hypertension, ntCTX	29
17	f	ntCTX	21
18	m	age >65, hypertension, targetedCTX	73
19	f	targetedCTX	26
20	m	ntCTX	21
21	f	age >65, ntCTX, NSAID	24
22	m	-	15
23	m	age >65, diabetes, hypertension, bisphosphonates	13
24	f	carcinoid syndrom, ntCTX	66
25	m	age >65, ntCTX	21
26	f	hypertension	8
27	m	age >65, hypertension, CVD	7
28	m	age >65, hypertension	34
29	f	ntCTX, targetedCTX	39
30	m	hypertension, CVD	0
31	m	hypertension, BMI >30	13
32	m	age >65, hypertension, CVD, targetedCTX	22
33	m	hypertension, CVD	0
34	m	bisphosphonates	4
35	f	-	0
36	m	ntCTX	17
37	f	bishosphonates, ntCTX, targetedCTX,	6
38	m	age >65, bisphosphonates, NSAID, targetedCTX	28
39	m	bisphosphonates, hypertension, s/p nephrectomy	15

CVD cardio-vascular-disease, NSAID >6 mo chronic intake of non-steroidal anti-inflammatory drugs, BMI body-mass-index, ntCTX chemo-therapy with known clinically relevant nephrotoxicity (e.g. platin), targetedCTX (no acute, but potentially indirect nephrotoxic potential of VEGF, mTOR or neoangiogenesis inhibitors)

For patients without individual dose estimates for previous PRRT, a replacement dose assuming 0.7 Gy/GBq ^{177}Lu-DOTATOC, 2.1 Gy/GBq ^{90}Y-DOTATOC or 1.6 Gy /GBq ^{213}Bi-DOTATOC was used.

Supplement Table 3: Sum activity of ^{225}Ac-DOTATOC, fractioning and follow-up period

**Pt.-No.***	**Ac-225 sum [MBq]**	1. AcPRRT [MBq]	*IBC [mo]*	2. AcPRRT [MBq]	*IBC [mo]*	3. AcPRRT [MBq]	*IBC [mo]*	4. AcPRRT [MBq]	*IBC [mo]*	5. AcPRRT [MBq]	**Follow-up [mo]**
**22**	**6**	6									**3**
**19**	**7**	7									**>83**
**32**	**10**	10									**5**
**12**	**18**	12	*2*	6							**20**
**21**	**24**	12	*14*	12							**24**
**28**	**58**	18	*4*	40							**4**
**29**	**58**	18	*4*	40							**12**
**7**	**38**	19	*2*	19							**73**
**10**	**19**	19									**>83**
**20**	**19**	19									**29**
**26**	**38**	19	*6*	19							**61**
**30**	**74**	19	*6*	19	*6*	19	*6*	19			**>73**
**36**	**37**	19	*2*	19							**4**
**38**	**38**	19	*6*	19							**20**
**39**	**57**	19	*4*	19	*6*	19					**44**
**4**	**20**	20									**15**
**5**	**40**	20	*4*	20							**15**
**15**	**20**	20									**13**
**18**	**20**	20									**25**
**25**	**91**	20	*4*	20	*4*	13	*6*	20	*8*	19	**27**
**16**	**24**	24									**1**
**6**	**75**	25	*4*	25	*4*	25					**14**
**11**	**50**	25	*4*	25							**42**
**13**	**74**	25	*4*	25	*6*	24					**11**
**33**	**25**	25									**7**
**34**	**25**	25									**>77**
**35**	**49**	25	*4*	24							**69**
**17**	**49**	30	*4*	19							**10**
**14**	**59**	33	*2*	26							**14**
**2**	**35**	35									**4**
**31**	**60**	35	*4*	25							**55**
**8**	**80**	40	*4*	40							**67**
**23**	**40**	40									**69**
**1**	**50**	50									**3**
**9**	**70**	50	*4*	20							**>93**
**27**	**87**	50	*3*	19	*3*	16					**18**
**37**	**70**	50	*4*	20							**60**
**3**	**95**	55	*4*	40							**35**
**24**	**60**	60									**1**

IBC Interval between cycles

* *sorted by 1st cycle treatment radioactivity*
