# Supplementary figures and images for: Dosing 225Ac-DOTATOC in patients with somatostatin-receptor-positive solid tumors: 5-year follow-up of hematological and renal toxicity
Source: Eur J Nucl Med Mol Imaging. 2021 Aug 26;49(1):54–63. doi: 10.1007/s00259-021-05474-1 (PMC8712294; doi:10.1007/s00259-021-05474-1)

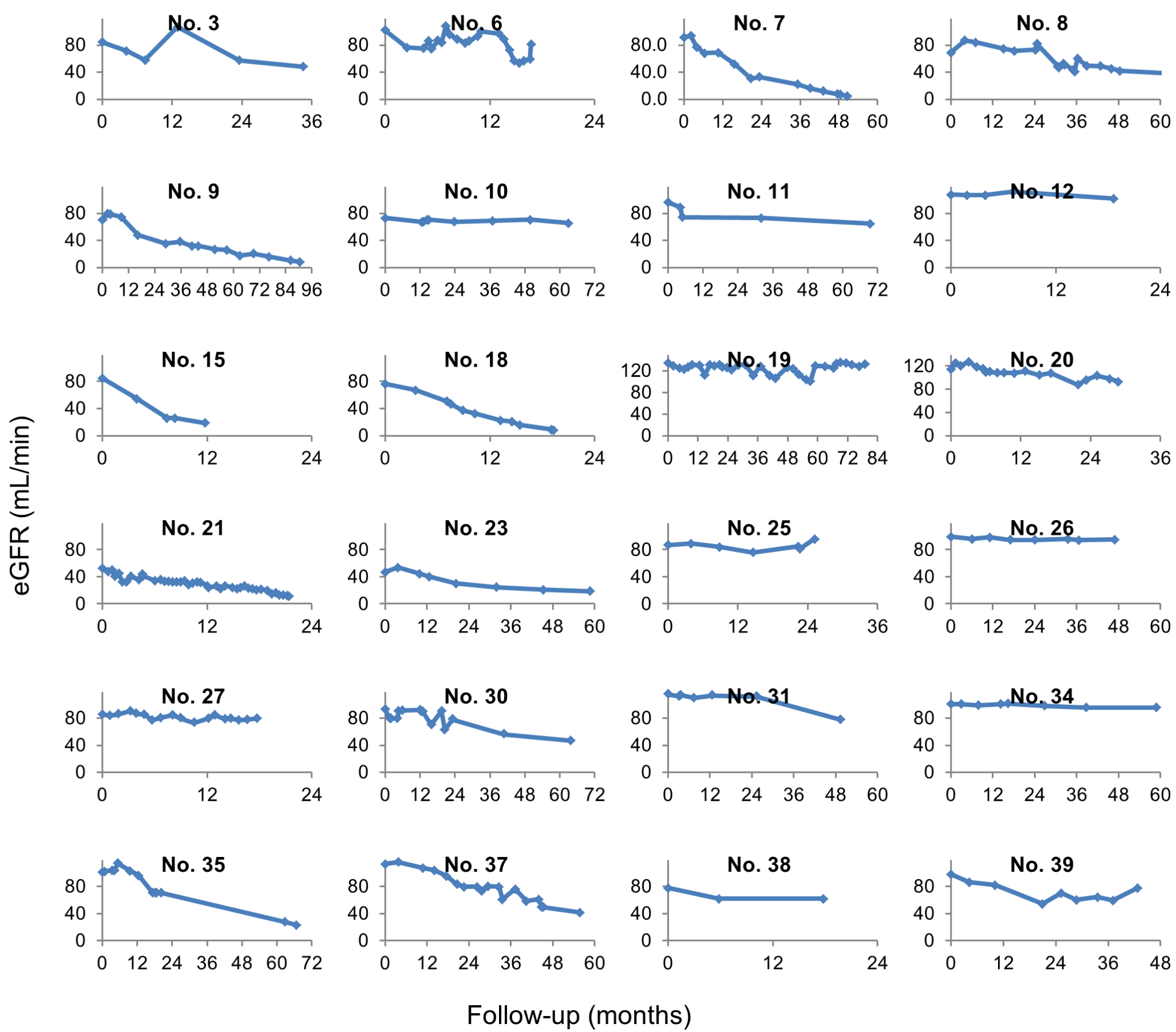

Supplement: Supplementary file 2 — (PNG 531 kb) [file 259_2021_5474_Fig6_ESM.png]

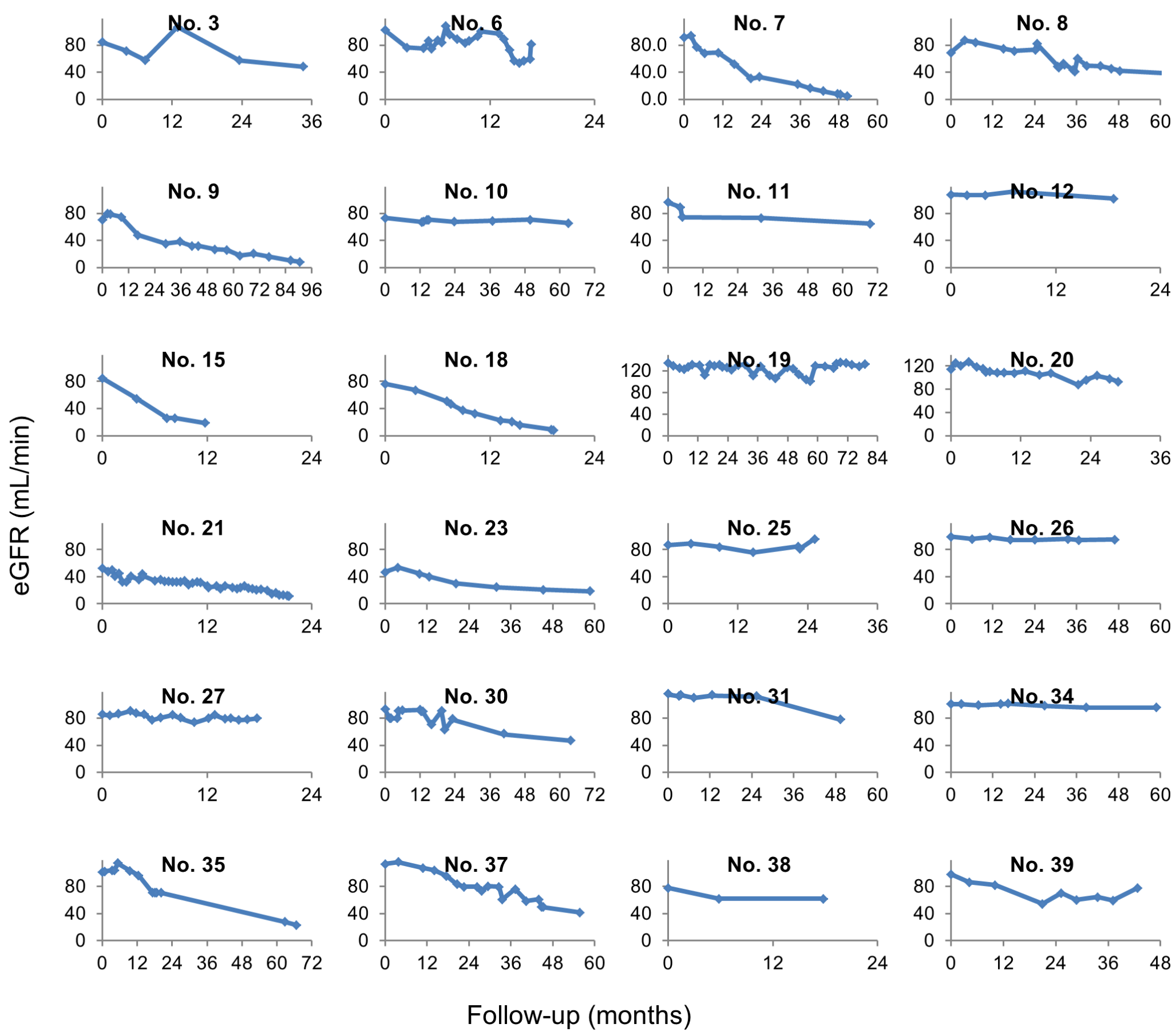

Supplement: Supplementary file 3 — High resolution image (TIF 780 kb) [file 259_2021_5474_MOESM2_ESM.tif]
